# Acetylcholinesterase Immobilized on Magnetic Beads for Pesticides Detection: Application to Olive Oil Analysis

**DOI:** 10.3390/s120607893

**Published:** 2012-06-08

**Authors:** Najwa Ben Oujji, Idriss Bakas, Georges Istamboulié, Ihya Ait-Ichou, Elhabib Ait-Addi, Régis Rouillon, Thierry Noguer

**Affiliations:** 1 IMAGES Laboratory, University of Perpignan, IMAGES EA4218, Building S 52 Av Paul Alduy, 66860 Perpignan Cedex, France; E-Mails: najwa_benoujji@hotmail.com (N.B.O.); idriss_salame@hotmail.com (I.B.); gistamboulie@yahoo.fr (G.I.); rouillon@univ-perp.fr (R.R.); 2 AQUAMAR Laboratory, Photocatalysis and Environment Team, Department of Chemistry, Faculty of Science, University Ibn Zohr, BP 8106 Cité Dakhla, Agadir, Morocco; E-Mails: ihya.aitichou@gmail.com (I.A.-I.); h.aitaddi@esta.ac.ma (E.A.-A.)

**Keywords:** electric eel acetylcholinesterase, organophosphorus insecticides, magnetic microbeads, olive oil

## Abstract

This work presents the development of bioassays and biosensors for the detection of insecticides widely used in the treatment of olive trees. The systems are based on the covalent immobilisation of acetylcholinesterase on magnetic microbeads using either colorimetry or amperometry as detection technique. The magnetic beads were immobilised on screen-printed electrodes or microtitration plates and tested using standard solutions and real samples. The developed devices showed good analytical performances with limits of detection much lower than the maximum residue limit tolerated by international regulations, as well as a good reproducibility and stability.

## 1. Introduction

Olive cultivation is widespread throughout the Mediterranean region and is important for the rural economy, local heritage and environment [1]. To ensure crop protection, the use of pesticides is often required for blocking attacks of pests and diseases, as well as presence of weeds. The olive fruit fly *Bactrocera oleae* is the most serious pest of olives in the Mediterranean countries, causing economic losses reaching up to 15% of olive production [2]. Insecticide treatments are applied every year to control the fly population, mainly based on pesticides belonging to the organophosphates class. These chemicals can persist to the harvest stage and are likely to contaminate olive oil. Therefore, both European Union and the Codex Alimentarius Commission of the Food and Agriculture Organization of the United Nations (FAO) have established maximum pesticide residues limits (MRLs) for olives and olive oil [3,4].

Conventional methods of detection of organophosphate pesticides rely on an analysis by gas chromatography with specific detection. Although these techniques are very powerful and can detect very low concentrations, they are still very expensive and require highly skilled personnel, expensive purification steps and specialized major equipment [5]. In the last decades, new technologies based on biological detection systems have emerged. Among these techniques, biosensors have been shown to be very promising due to their simplicity and cost effectiveness compared to conventional techniques. Biosensors based on the inhibition of acetylcholinesterase (AChE) have been intensively studied in the aim of detecting organophosphorus insecticides [6]. Cholinesterases are important enzymes present in vertebrates and insects, which hydrolyze the neurotransmitter acetylcholine in the nervous system [7]. Organophosphorus pesticides are esters, amides or thiol derivatives of phosphoric acid esters. They primarily exist in the thionate form which is stable, but not very active. Activation occurs during metabolic oxidation into the biologically active oxon form, which is much less stable [8]. These insecticides act by phosphorylation of the serine located in the catalytic site of AChE, they can be considered as pseudo-substrates [9]. As this phosphorylation is very difficult to reverse, organophosphates are considered as irreversible inhibitors. This irreversibility is probably the main problem related to AChE-based biosensors, because of the difficulty in performing multiple assays using the same sensor [6]. Several methods have been investigated to overcome this problem, including mainly reactivation using oximes [10] and original immobilisation techniques.

**Figure 1.**
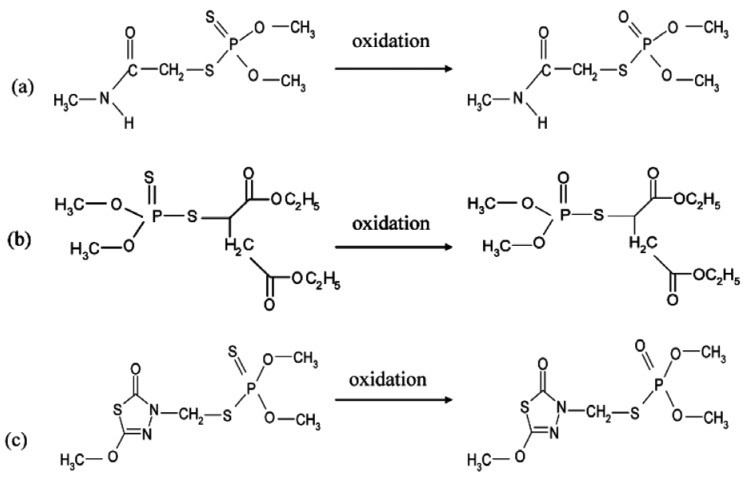
Structures of organophosphates and their oxidized forms used in this study: (**a**) dimethoate and his oxon form omethoate; (**b**) malathion and malaoxon; (**c**) methidathion and methidathion oxide.

Among these immobilisation methods, magnetic particles have recently gained a great attention due to their potential for providing control of electrochemical processes [11] and creating magneto-switchable devices [12,13]. Immobilization of enzymes, antibodies, oligonucleotides, and other biologically active compounds onto magnetic nanoparticles platforms is a key element in using these structures for biosensing purposes. Fabricating biofunctionalized magnetic materials containing a high amount of the biological element with high activity and stability is essential for the design of robust sensors that take advantage of the magnetic capabilities. The different routes for the fabrication of biofunctionalized magnetic nanoparticles include traditional methods such as covalent binding, adsorption, specific affinity interactions, and entrapment in porous surface layers [14]. Immobilisation of acetylcholinesterase on magnetic microbeads was already described in the literature, based on nickel-histidine affinity [6,15]. In this work we propose an immobilisation method that can be applied to the native acetylcholinesterase from electric eel, based on covalent coupling on magnetic microbeads. This method allows designing cheaper biosensors allowing the detection of insecticides in olive oil (Figure 1). The modified beads have been used either in bioassay or in biosensor configurations, based respectively on spectrophotometric or amperometric detection methods.

## 2. Experimental Section

### 2.1. Chemicals and Stock Solutions

AChE (EC 3.1.1.7) from electric eel (EE) (Type V-S, 1,000 U/mg) was purchased from Sigma-Aldrich (St Quentin-Fallavier, France). Acetylthiocholine chloride (ATChCl), acetylthiocholine iodide (ATChI) and 5,5-dithiobis(2-nitrobenzoic acid) (DTNB-Ellman's reagent) were provided by Sigma. In order to minimize hydrolysis, ATChCl and ATChI solutions were prepared daily in 0.9% NaCl (Sigma-Aldrich) solution. Stock solutions of enzymes and DTNB were prepared in 0.1 M phosphate buffer (Na_2_HPO_4_/KH_2_PO_4_, Sigma-Aldrich) at pH 7. The organophosphorus insecticides malaoxon, omethoate and methidathion were purchased from Dr. Ehrenstorfer (Augsburg, Germany). Pesticide stock solutions (concentration 10^−3^ M) were prepared in acetonitrile (Sigma) and stored at 4 °C, working pesticide solutions were prepared daily in distilled water by dilution of the stock solution. The oxidation of methidathion was achieved using N-bromosuccinimide provided by Sigma-Aldrich. The glutaraldehyde used for activation of magnetic beads was also purchased from Sigma-Aldrich. Carbon (Electrodag 423SS) and silver/silver chloride (Electrodag 418SS) inks were obtained from Acheson (Plymouth, UK). Cobalt phtalocyanine-modified carbon paste was purchased from Gwent Electronic Materials, Ltd. (Gwent, UK). Poly(vinyl)chloride (PVC) sheets (200 mm × 100 mm × 0.5 mm), supplied by SKK (Denzlingen, Germany), were used as support for the screen-printed electrodes. A glycerophthalic paint (Astral, France) was used as insulating layer.

### 2.2. Apparatus

Spectrophotometric measurements were performed using a Hewlett Packard diode array 8451A spectrophotometer. Colorimetric measurements on PS-microtiter plates, U form (Greiner, Germany) were performed with a Labsystems Multiskan EX microtiter plate reader (Thermo Life Sciences, France). Amperometric measurements were carried out with a 641VA potentiostat (Metrohm, Switzerland), connected to a BD40 (Kipp & Zonen, The Netherlands) flatbed recorder.

Screen-printed electrodes were produced using a semi-automatic DEK248 printing machine according to a procedure previously described [15], but in a three-electrode configuration. The working electrode was a 4 mm-diameter disk, the auxiliary electrode was a 16 mm × 1.5 mm curved line and the Ag/AgCl pseudo-reference electrode was a 5 mm × 1.5 mm straight track. For experiments with magnetic beads, a small 4 mm-diameter magnet was placed on the backside of the working electrode to magnetically attach the enzyme-functionalised beads to the electrode surface.

### 2.3. Determination of Acetylcholinesterase Activity

The activity of AChE was measured spectrophotometrically by monitoring at 412 nm the appearance of thionitrobenzoate resulting from the reaction of DTNB with thiocholine, the product of the enzymatic hydrolysis of acetylthiocholine substrate, according to the procedure described by Ellman *et al.* [16]. This method is based on the use of a synthetic substrate: acetylthiocholine, whose hydrolysis liberates thiocholine and acetic acid according to the reaction:

The thiocholine reacts with 5,5′-dithiobis-(2-nitrobenzoic acid) (DTNB) yielding a yellow complex absorbing at 412 nm (ε = 1.36 × 10^4^ M^−1^cm^−1^).

### 2.4. Determination of the Inhibition Constant ki

The mechanism of inhibition of AChE by organophosphate compounds is well-known [17]. The inhibitor phosphorylates a serine located in the active site and the inhibition can be considered as irreversible in the first 30 min [18]:

$$\text{E}+\text{PX}\overset{{\text{K}}_{\text{d}}}{↔}\text{E}-\text{PX}\overset{{\text{k}}_{2}}{\to }\text{EP}+\text{X}$$

With E = enzyme, PX = organophosphate and X = leaving group. This scheme can be simplified with the bimolecular constant k_i_ = k_2_/K_d_:

$$\text{E}+\text{PX}\overset{{\text{k}}_{\text{i}}}{\to }\text{EP}+\text{X}$$

To follow the inhibition, the enzyme was incubated with the pesticide during different periods of time, at 30 °C in 0.1 M phosphate buffer, pH 7. The change in remaining free enzyme concentration [E]/[E_0_] with time was estimated by sampling aliquots at various times and recording the remaining activity in the presence of 1mM acetylthiocholine [19].

The experimental procedure was as follows: 300 μL of 2.5 × 10^−3^ M DTNB and 100 μL of 0.01 M ATChI were added to 500 μL of 0.1 M phosphate buffer at pH 7; then 100 μL of the enzyme-inhibitor solution were taken at fixed time intervals and added to the cell. The incubation times used to study EE-AChE inhibition were 0, 1, 3, 5, 7, 10, 15 and 20 min.

The residual activity of AChE was calculated by comparing the slope of obtained kinetics before and after inhibition. The graphs obtained by plotting log of residual activity *vs.* incubation time for each inhibitor showed a linear representation. The apparent reaction rate k_obs_ (min^−1^) were obtained by measuring the slope of this straight line. Plotting 1/k_obs_ *vs.* 1/[I] allowed calculating the inhibition constant k_i_, which corresponds to the reciprocal value of the obtained slope.

### 2.5. Immobilisation on Magnetic Nanoparticles by Covalent Coupling

Nickel magnetic beads with a diameter of 200 nm were activated according to the following steps [20]:

1. Oxidation of the beads: 60 mg of magnetic beads were stirred for 4 h in 1 mL of 0.5 M sulfuric acid, and then washed twice with distilled water.
2. Functionalization with an amine group: 70 μL of 3-aminopropyltriethoxysilane were added to the beads previously poured in 100 mL of ethanol and ultrasonicated during 5 min, the suspension was kept under mechanical stirring overnight and finally washed three times with ethanol and twice with distilled water.
3. Covalent coupling with glutaraldehyde: 30 μL of aminated beads were washed twice with 1 mL of 0.1 M pH 7 buffer. 820 μL of buffer, 100 μL of electric eel AChE (4.41 UI/mL) and 80 μL of a 25% glutaraldehyde solution were added to the beads and stirred during 30 min at room temperature.
4. 1 μL of the obtained enzyme-linked beads suspension was placed either on the surface of the working electrode, beforehand fitted with a 4 mm-diameter magnet (amperometric detection), or in each well of the microplate (colorimetric detection).

### 2.6. Measurements

#### 2.6.1. Amperometric Measurements

The electrode was vertically immersed in a thermostated cell (30 °C) containing 10 mL phosphate buffer pH 7 under constant magnetic stirring (417 rpm). The applied potential was 100 mV *vs.* Ag/AgCl reference electrode, using cobalt phtalocyanine as mediator. The current intensity was recorded and, after current stabilisation, 1 mM ATCh (final concentration) was added in the cell. The measured signal corresponded to the difference of current intensity between the baseline and the plateau. The cell was washed with distilled water between measurements.

The pesticide detection was made in a three-step procedure as follows: first, the initial response of the electrode to 1 mM ATCh was recorded three times, then the electrode was incubated in a solution containing a known concentration of insecticide, and finally the residual response of the electrode was recorded again. Electrodes were thoroughly washed with distilled water between each measurement. The percentage of the inhibition was correlated with the insecticide concentration, the inhibition rate was calculated according to the following formula: I (%) = [(I_0_ − I)/I_0_]100, I and I_0_ being respectively the current after the and before inhibition.

#### 2.6.2. Colorimetric Measurements

Two hundred μL of phosphate buffer pH 8 were added in each well containing AChE-modified magnetic beads suspension in order to equilibrate the enzyme. After removal of the liquid using the Adem-Mag96 [21] 200 μL of phosphate buffer containing 2 mM ACTh-I and 6% DTNB were added and the microplate was incubated for 30 min under constant orbital stirring (300 rpm). The absorbance was then measured at 405 nm using 100 μL of the solution taken from each well. Inhibition experiments were performed by incubating the magnetic beads (1 μL) with 100 μL of different concentrations of pesticide during 10 min. The measurement procedure was the same as described above.

### 2.7. Extraction Procedure

The determination of the three pesticides in natural oil samples was performed after a simple liquid-liquid extraction procedure. The extraction was performed using 10 mL of olive oil previously spiked with pesticides at final concentrations of 10^−2^ M for omethoate and methidathion, and 10^−3^ M for malaoxon. The mixture was heated at 50 °C for 30 min, and 500 μL of this olive oil was added to 400 μL of acetonitrile and 100 μL of dichloromethane. The mixture was centrifuged at 13,400 rpm for 90 s and the resulting supernatant was recovered and used as a pesticide mother solution, other pesticide solutions were prepared by diluting this mother solution.

### 2.8. Oxidation of Methidathion

In this study we have focused on the detection of oxidized forms of each pesticide, which are less stable but more toxic than the normal forms. The oxidized forms of dimethoate and malathion, respectively called omethoate and malaoxon are commercially available, but in the case of methidathion an oxidation step must be carried out using *N*-bromosuccinimide (NBS). The efficiency of this oxidation step was controlled using reverse-phase HPLC, it was shown that 3 × 10^−5^ M NBS was sufficient for the total oxidation of a 10^−5^ M methidathion solution. The effect of NBS on AChE was investigated to ensure that the enzyme is not affected by the oxidizing agent, it was shown that in assays conditions NBS did not inhibit AChE.

## 3. Results and Discussion

### 3.1. Determination of the Inhibition Constant ki

The inhibition constants k_i_ were calculated by performing enzyme kinetic measurements using different pesticide concentrations and by varying the incubation time of the enzyme with the pesticide. The residual enzyme activity was determined according to the Ellman's spectrophotometric method as described previously. The inhibition constant k_i_ is proportional to the affinity of the pesticide for the enzyme and its inhibitory power. k_i_ is therefore a fundamental parameter to compare the inhibitory potency of insecticides and assess the sensitivity of the enzymes studied. The constants k_i_ presented in Table 1 have been determined by studying the residual activity of the enzyme after contact with different concentrations of inhibitor for a given incubation time. This value of k_i_ shows that the acetylcholinesterase of electric eel is highly sensitive to malaoxon and methidathion but very weakly sensitive to omethoate.

### 3.2. Detection of Insecticides in Microplate Assays

#### 3.2.1. Optimisation of the Reaction Time

The time of reaction of acetylthiocholine with immobilized AChE was studied by measuring every 5 min the appearance of the yellow complex at 412 nm. It was shown that the reaction was completed after 30 min in absence of pesticide; this time was therefore used in inhibition measurements.

#### 3.2.2. Detection of Organophosphates by Colorimetric Method

**Figure 2.**
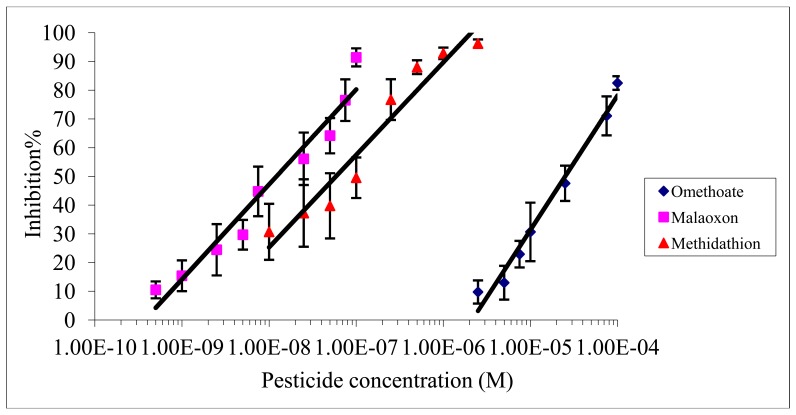
Inhibition effect of omethoate, methidathion and malaoxon on electric eel AChE immobilized on magnetic beads, measurement by colorimetric method.

The inhibition effect of malaoxon, methidathion and omethoate on Electric eel AChE was studied using an incubation time of 10 min and a measurement time of 30 min. Figure 2 shows the inhibition rates obtained with each pesticide as a function of the concentration used.

As expected from the values of inhibition constants (Table 1), the inhibitory effect of omethoate was the weaker and malaoxon was the more effective inhibitor. The limit of detection (LOD) and the IC50 were calculated as the pesticide concentration inducing respectively 10% and 50% inhibition. The IC10 and IC50 obtained for each pesticide are summarized in Table 2.

Beside these results, the microplate assay showed a good reproducibility with a stable response, this detection method has the advantage of saving time as it allows analysing 96 samples in a single assay. Furthermore, due to the small volumes used, it allows one to minimize the use of reagents, however this characteristic can be considered also as a disadvantage because of the difficulty to maintain good reproducibility using small volumes.

### 3.3. Detection of Organophosphates by an Amperometric Method

The operational stability of biosensors based on AChE covalently bound to magnetic beads was tested to ensure that any decrease in the signal was related to enzyme inactivation and not to enzyme leakage. This parameter was estimated by repetitive measurements of the response of a single electrode to 1 mM ATCh, with intermediate rinses with distilled water. In these conditions, the designed sensors showed a very good stability for at least 10 consecutive measurements with a relative standard deviation (R.S.D) in order of 3% (300 ± 10 nA). The reproducibility of the inhibition was estimated by measuring six times the inhibition percentage by 3 × 10^−8^ of oxidized methidathion, the R.S.D of the inhibition determination was 4% (217 ± 10).

**Figure 3.**
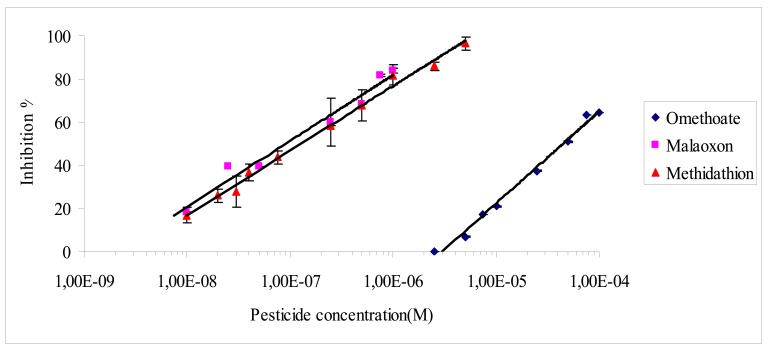
Inhibition effect of omethoate, methidathion and malaoxon on the amperometric biosensor based on electric eel AChE covalently immobilized on magnetic microbeads.

The incubation time between the biosensor and pesticides was also tested. It was observed that an incubation time of 10 min was sufficient to reach the maximum inhibition rate whatever the pesticide concentration used. This incubation time was thus selected and used for all inhibition assays. Figure 3 presents the inhibition effect of the three pesticides on the biosensor. As expected the biosensor displays a very low limit of detection for malaoxon and methidathion, while omethoate is detected with a very low sensitivity (Figure 3). The IC_10_ and IC_50_ values obtained for each pesticide are summarized in Table 2.

### 3.4. Application to Real Samples

The performance of the biosensors was tested using olive oil samples previously spiked with known concentrations of pesticides. In order to measure the efficiency of the extraction procedure, three olive oil samples were spiked to obtain 10^−2^ M of omethoate as final concentration and extracted according to the method described in Section 2.7. The inhibition effect of the resulting extracts was measured after dilution in the working buffer, at a final concentration of 10^−4^ M. In these conditions, the average response of the sensors was 75 ± 7 nA, showing a good reproducibility of the extraction method. The recovery percentage for the three pesticides was calculated using the formula below:

Recovery%=%ofinhibition in real samples/%of inhibition in buffer×100

**Figure 4.**
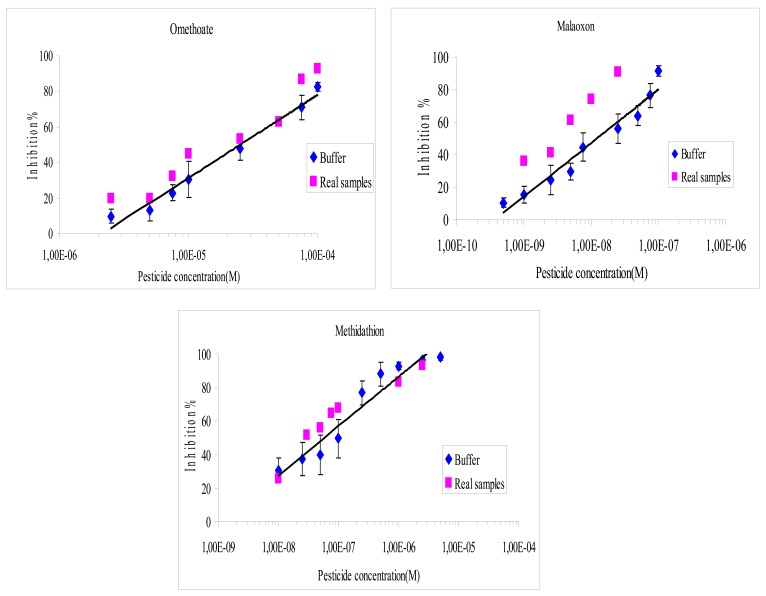
Comparison of inhibition effects of omethoate, methidathion and malaoxon in olive oil real samples and buffer, detection by optical method.

**Figure 5.**
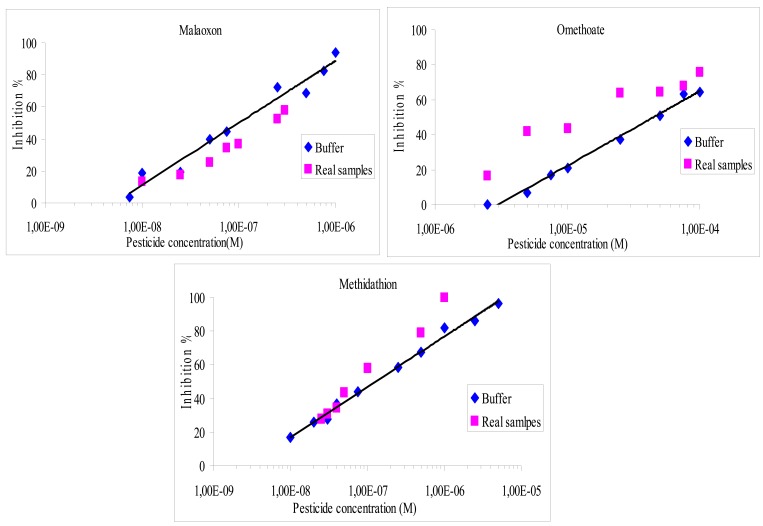
Comparison of inhibition effects of omethoate, methidathion and malaoxon in buffer or olive oil real samples, detection by amperometric method.

It was found that the recovery percentage for the three pesticides with both methods varies from 40% to 170%, as shown in Figures 4 and 5. The results showed a very satisfying correlation whatever the format used, *i.e.*, microplate assay (Figure 4) or biosensor (Figure 5).

Despite that, a small increase of the inhibition effect was observed whatever the pesticide studied, especially when using the microplate assay. These small discrepancies could be attributed to matrix effects. In order to evaluate these interferences, a blank assay was carried out using an olive oil sample that was previously extracted and then spiked to obtain 10^−2^ M of omethoate. It was found that the matrix effect was responsible of a 5.5% supplementary loss of the sensor response, which can explain the increase in inhibition ratios observed using real samples. Additional blank assays were performed to verify that the inhibition was not due to pesticides already present in the olive oil, these tests showed very low inhibition rates, lower than 4%. The inhibition effect of olive oil samples containing NBS has also been studied, it was shown that NBS did not affect the measurement.

## 4. Conclusions/Outlook

In this work we have developed and optimised the immobilisation of acetylcholinesterase on magnetic microbeads by covalent coupling. The modified beads have been used in two configurations, using either microplates or screen-printed electrodes supports. Depending on the configuration, colorimetry or amperometry have been applied as detection methods and the devices have been tested on standard and real samples for the determination of three pesticides commonly used for the treatment of olive trees: omethoate, malaoxon and methidathion. The results showed a good performance of the developed devices with reasonable limits of detection, as well as good reproducibility and stability. These last two criteria were however weaker for the colorimetric method due to the difficulty in handling small volumes in the microplate assay. On the other hand, this configuration had the advantage of being faster and more economical. This study highlighted the low sensitivity of the electric eel acetylcholinesterase for omethoate compared to malaoxon and oxidized methidathion, which lead to detection limits as low as 10^−10^ M. The application on the spiked olive oil samples showed very satisfactory results, comparable with those obtained using standard solutions.

## Figures and Tables

**Table 1.** Inhibition constants ki (μM^−1^·min^−1^) obtained for methidathion, malaoxon and omethoate.

	**Methidathion**	**Malaoxon**	**Omethoate**
**AChE-EE**	1.07	2.98	0.001

**Table 2.** The IC_50_ and IC_10_ values (M) obtained with the two detection methods with the three pesticides.

	**Colorimetric detection Pesticide concentration (M)**		**Amperometric detection Pesticide concentration (M)**	
	IC_10_	IC_50_	IC_10_	IC_50_
**Omethoate**	3.45E-06	2.65E-05	5.15E-06	4.46E-05
**Methidathion**	2.50E-09	5.80E-08	8.85E-09	1.36E-07
**Malaoxon**	8.00E-10	1.30E-08	9.40E-09	1.00E-07
